# Supplementary material for: Deficiency of IQCH causes male infertility in humans and mice
Source: eLife. 2024 Jul 19;12:RP88905. doi: 10.7554/eLife.88905 (PMC11259432; doi:10.7554/eLife.88905)
Supplement: Figure 1—source data 1. [file elife-88905-fig1-data1.docx]

**Figure 1—source data 1.** Primers for Sanger sequencing and Minigene.

| **Variant** | **Sequence** |
| --- | --- |
| Sanger sequencing primer of *IQCH* | F 5' GACATGGCAGATGCAGAATCTG 3' |
|  | R 5' TGAATTTAGAGCAAGTTCATCAGAGA 3' |
| Amplification primer of *IQCH* for minigene assay | F 5' TACGGGATCACCAGTCCGCCTTCCGGGTTCATGCCACTCTC 3' |
|  | R 5' TCACCAGATATCTGGCAAGAGCCTATGGCCCACACCCTAGGC 3' |
| Amplification primer of plasmid for minigene assay | F 5' TCTGAGTCACCTGGACAACC 3' |
|  | R 5' ATCTCAGTGGTATTTGTGAGC 3' |
