# Supplementary figures and images for: Deficiency of IQCH causes male infertility in humans and mice
Source: eLife. 2024 Jul 19;12:RP88905. doi: 10.7554/eLife.88905 (PMC11259432; doi:10.7554/eLife.88905)

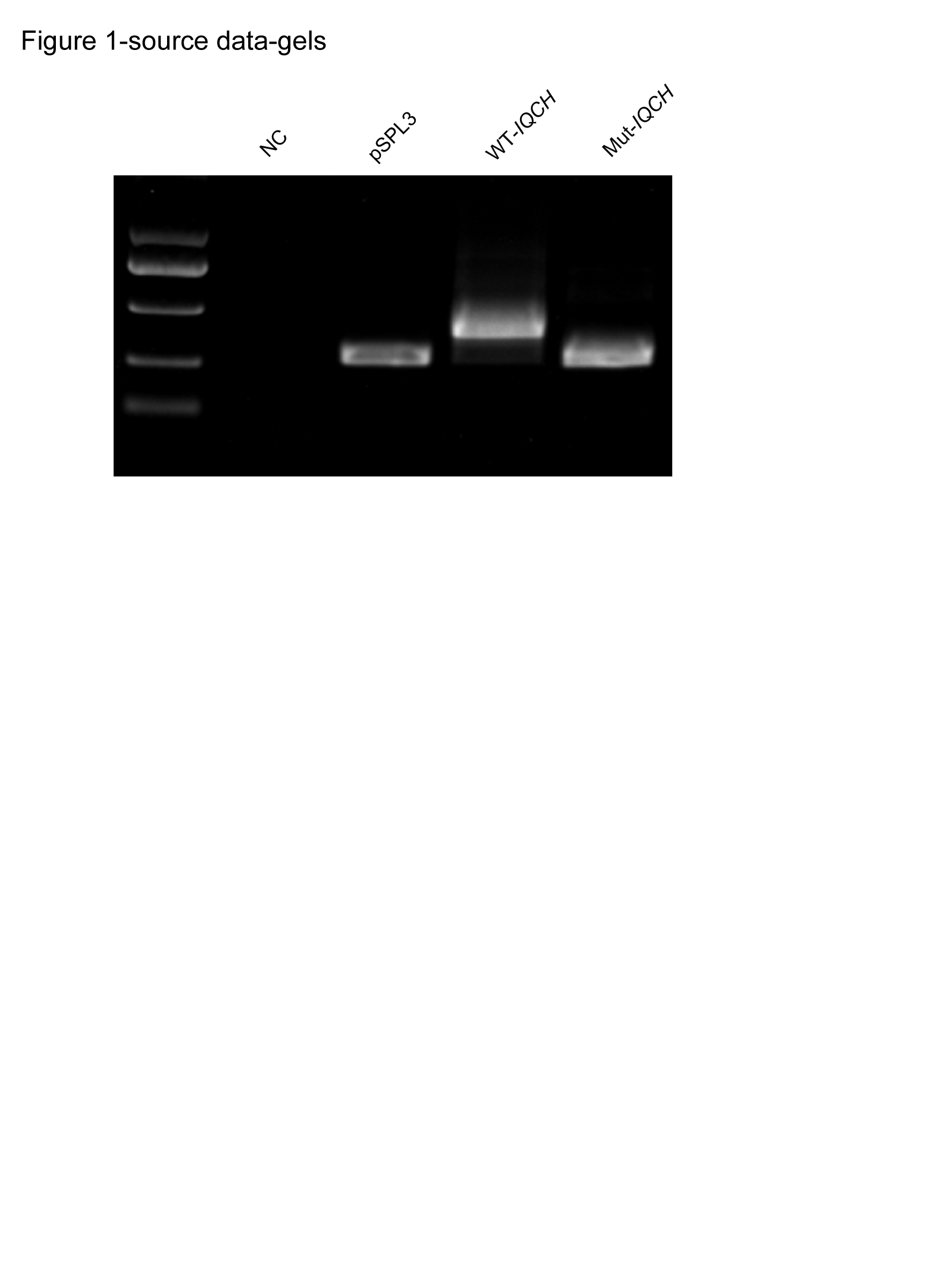

Supplement: Figure 1—source data 2. [file elife-88905-fig1-data2.zip › Figure1SourceData2/Figure 1-source data-gels.tif]

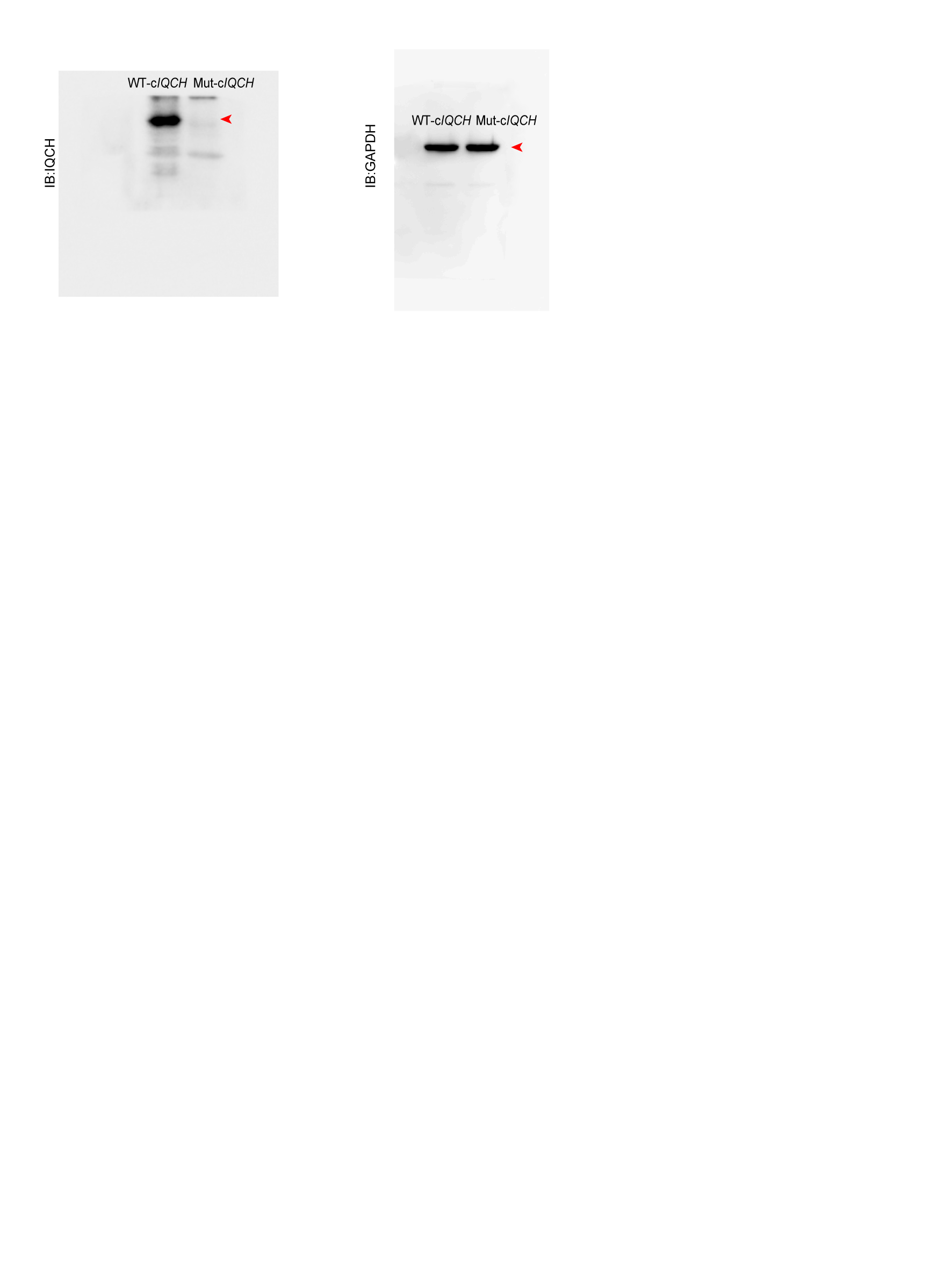

Supplement: Figure 1—source data 2. [file elife-88905-fig1-data2.zip › Figure1SourceData2/Figure 1-source data-blot.tif]

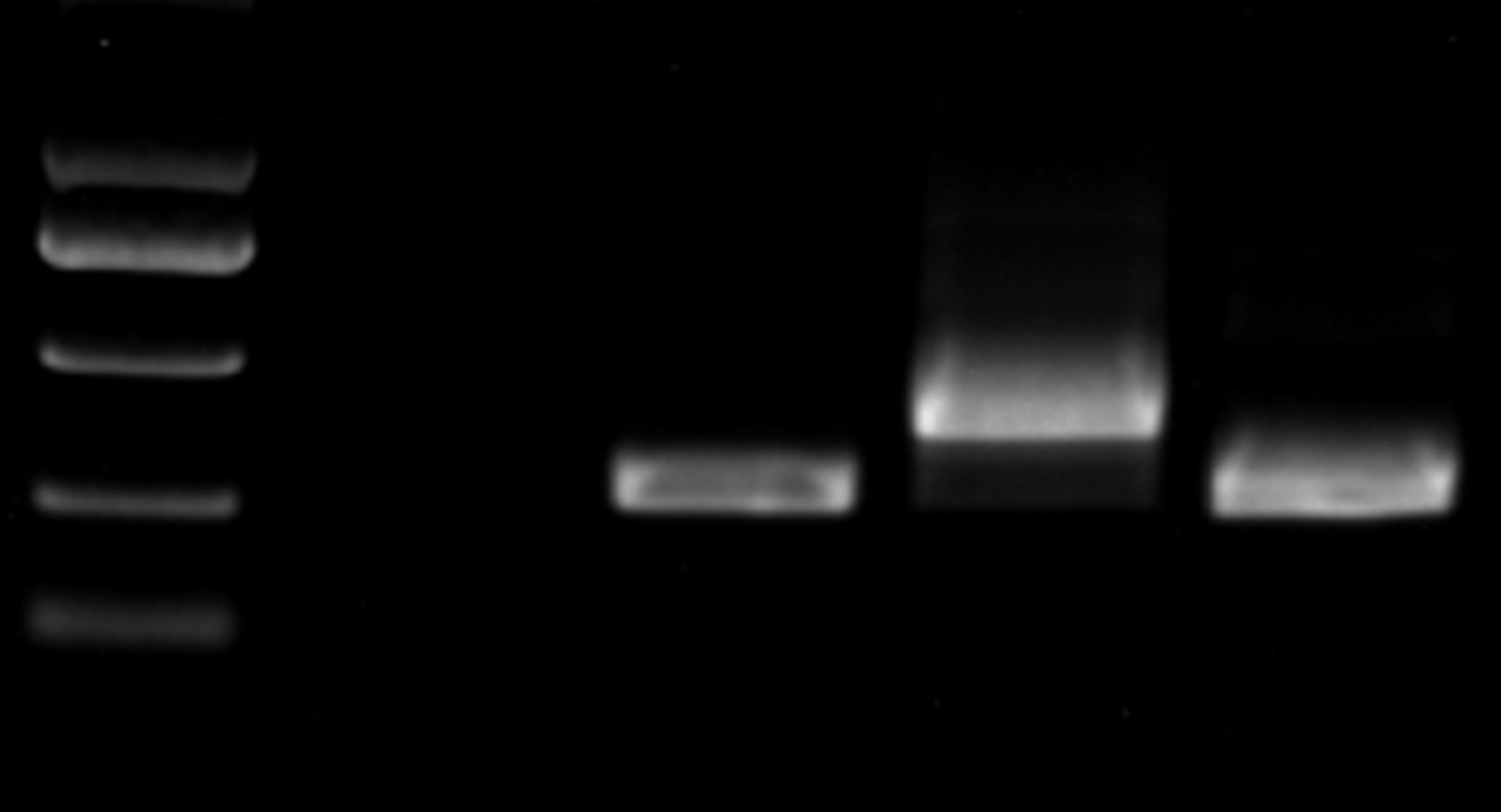

Supplement: Figure 1—source data 2. [file elife-88905-fig1-data2.zip › Figure1SourceData2/rawdata picture/data 1.pdf]

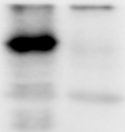

Supplement: Figure 1—source data 2. [file elife-88905-fig1-data2.zip › Figure1SourceData2/rawdata picture/data 2.pdf]

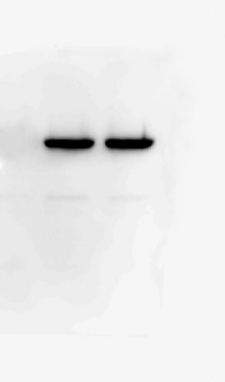

Supplement: Figure 1—source data 2. [file elife-88905-fig1-data2.zip › Figure1SourceData2/rawdata picture/data 3.pdf]

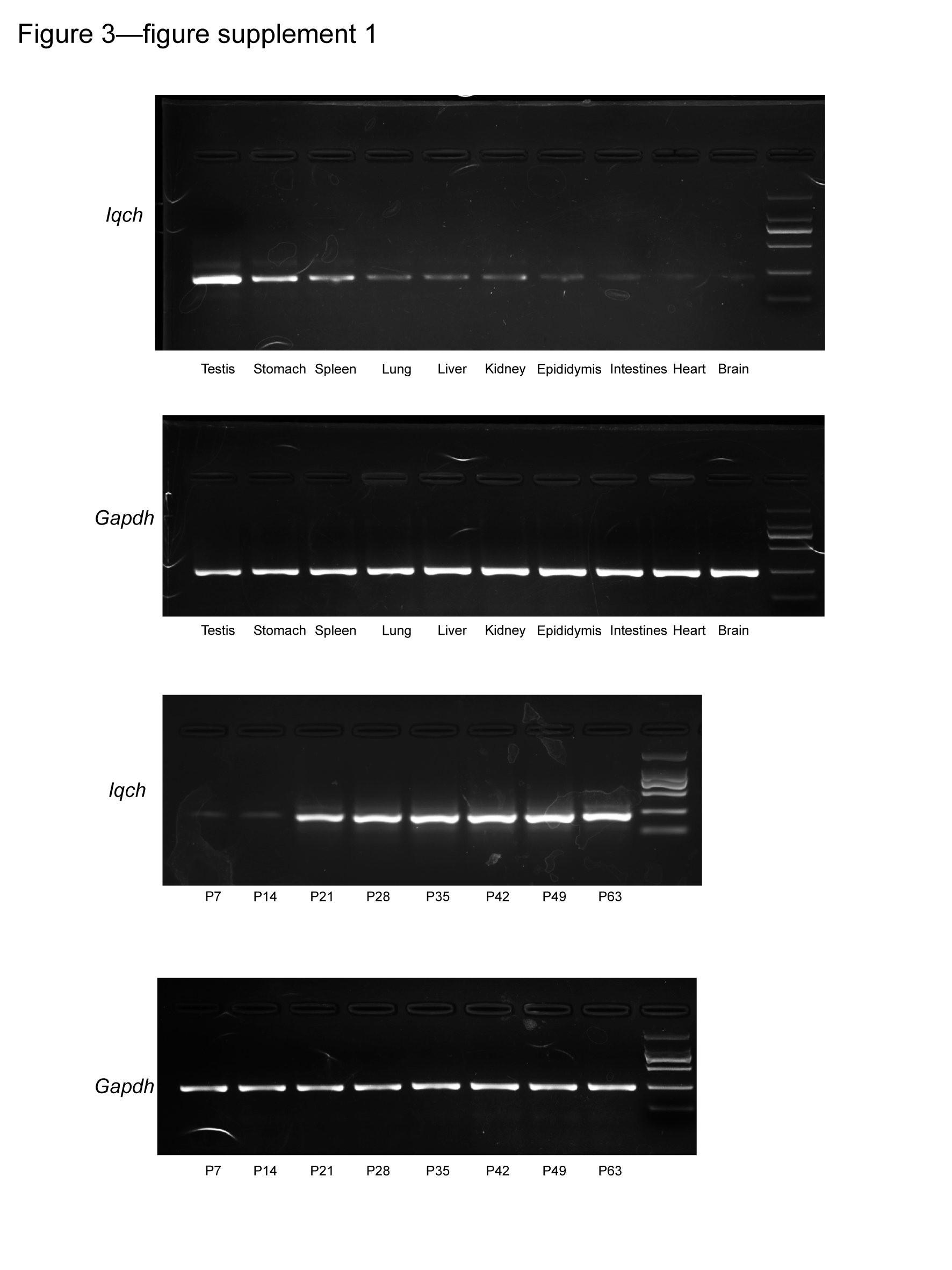

Supplement: Figure 3—figure supplement 1—source data 1. [file elife-88905-fig3-figsupp1-data1.zip › Figure3S1SourceData1/Figure 3-figure supplement 1-source data-gels.tif]

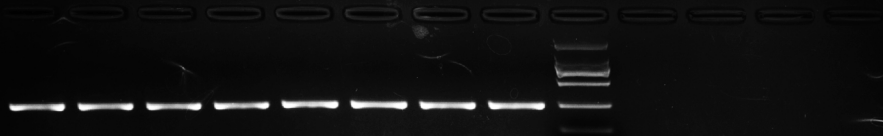

Supplement: Figure 3—figure supplement 1—source data 1. [file elife-88905-fig3-figsupp1-data1.zip › Figure3S1SourceData1/rawdata picture/data 4.pdf]

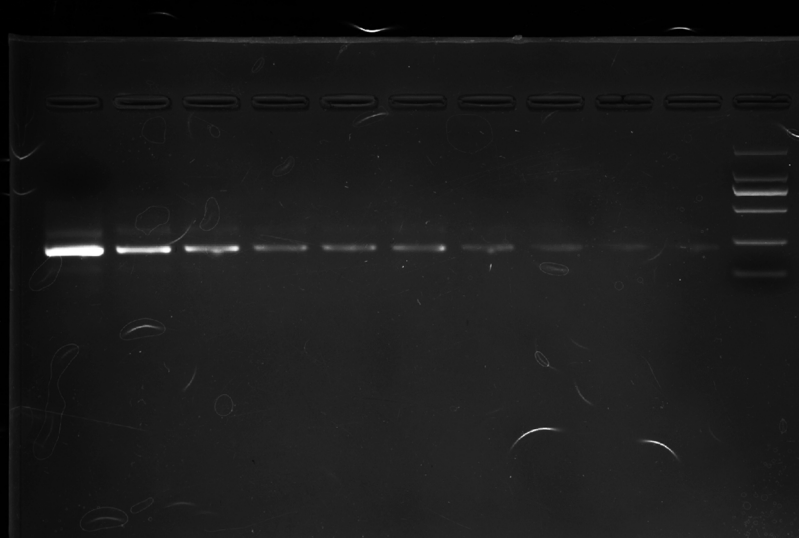

Supplement: Figure 3—figure supplement 1—source data 1. [file elife-88905-fig3-figsupp1-data1.zip › Figure3S1SourceData1/rawdata picture/data 1.pdf]

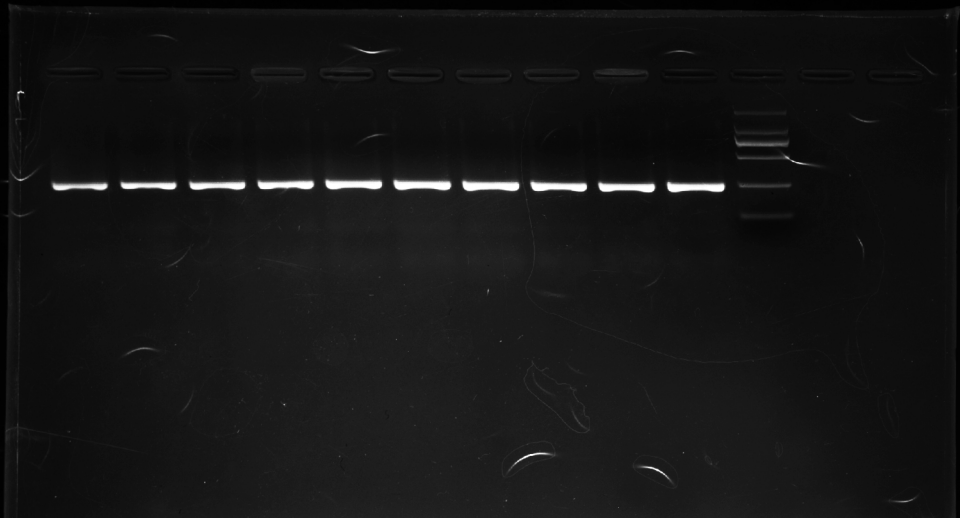

Supplement: Figure 3—figure supplement 1—source data 1. [file elife-88905-fig3-figsupp1-data1.zip › Figure3S1SourceData1/rawdata picture/data 2.pdf]

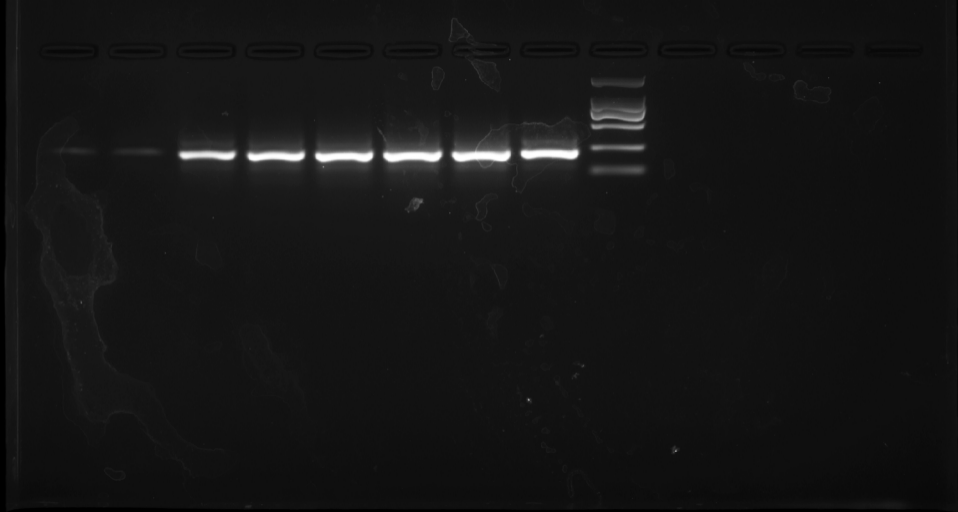

Supplement: Figure 3—figure supplement 1—source data 1. [file elife-88905-fig3-figsupp1-data1.zip › Figure3S1SourceData1/rawdata picture/data 3.pdf]

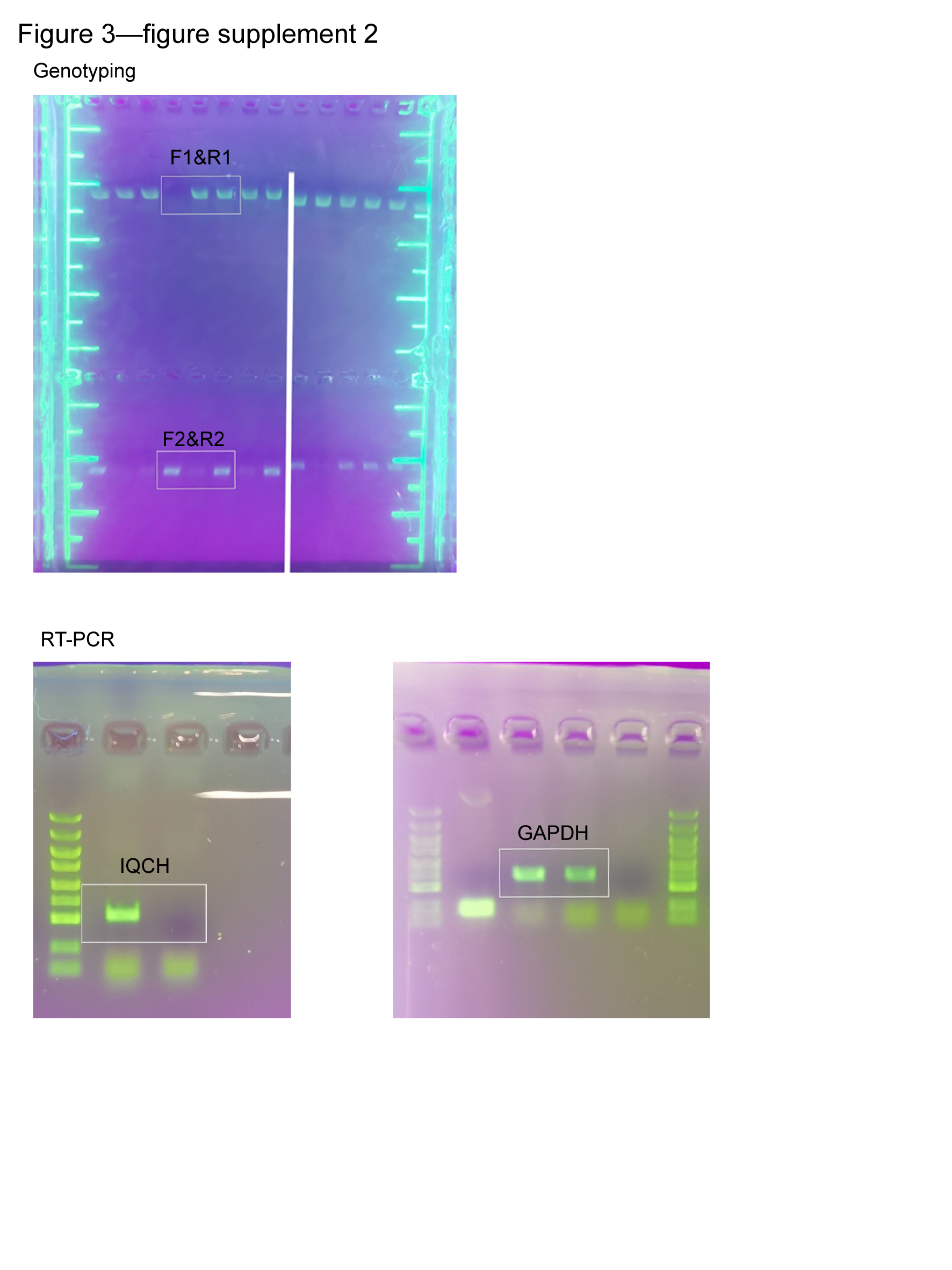

Supplement: Figure 3—figure supplement 2—source data 1. [file elife-88905-fig3-figsupp2-data1.zip › Figure3S2SourceData1/Figure 3-figure supplement 2-source data-gels.tif]

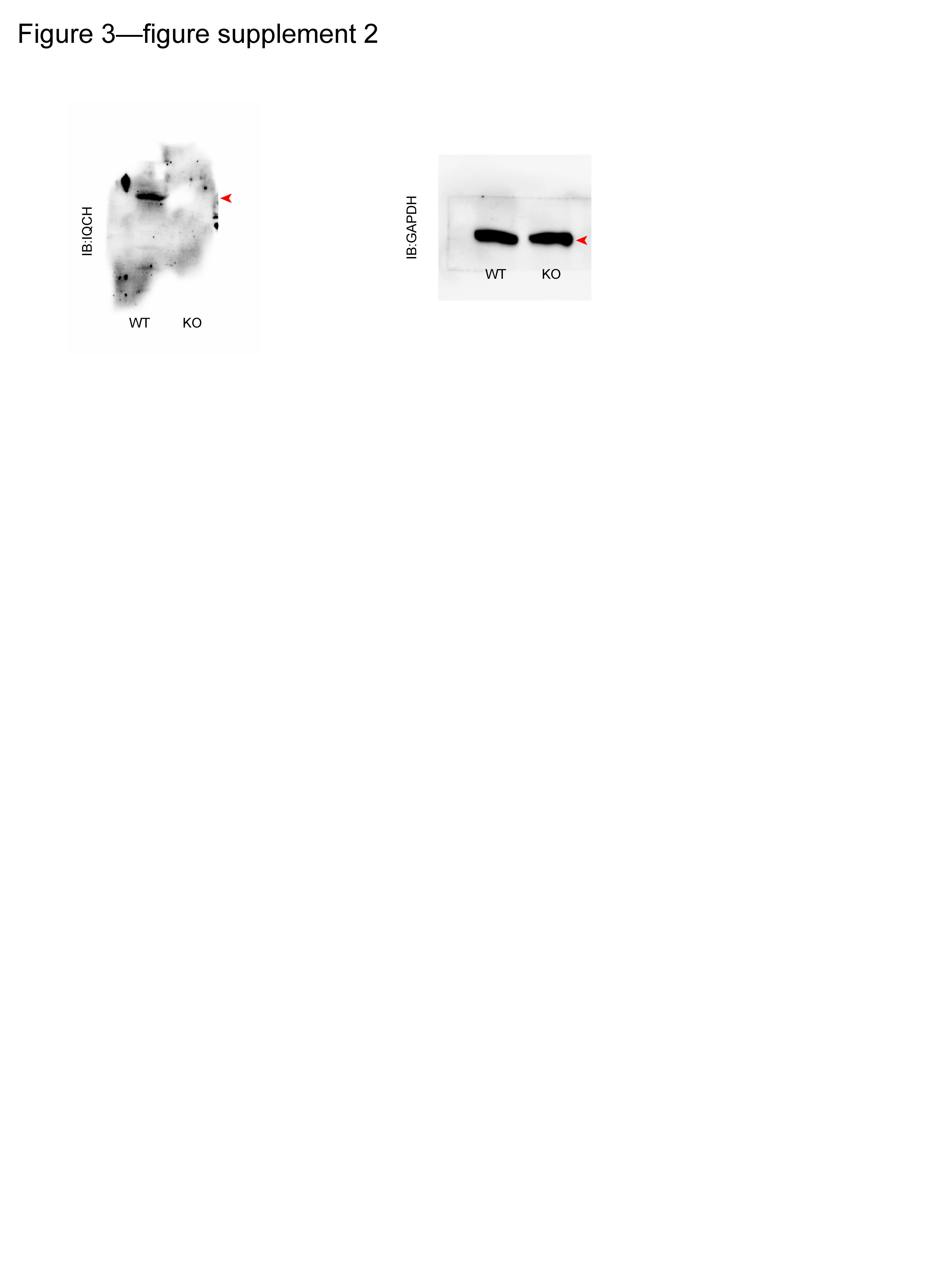

Supplement: Figure 3—figure supplement 2—source data 1. [file elife-88905-fig3-figsupp2-data1.zip › Figure3S2SourceData1/Figure 3-figure supplement 2-source data-blots_┬¡1.png]

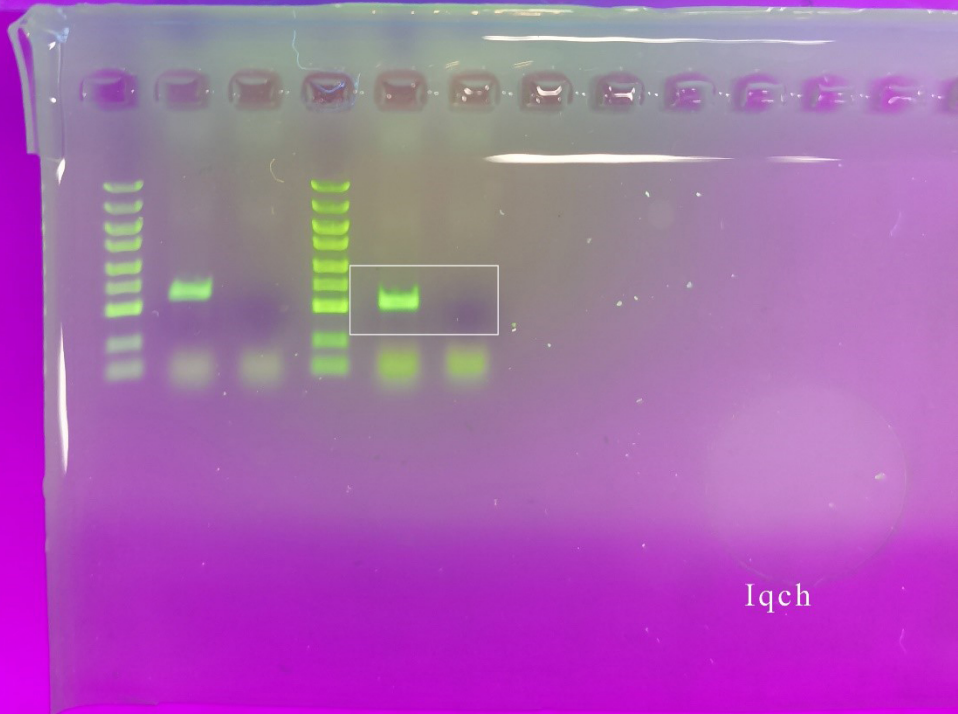

Iqch

Supplement: Figure 3—figure supplement 2—source data 1. [file elife-88905-fig3-figsupp2-data1.zip › Figure3S2SourceData1/rawdata picture/data 4.pdf]

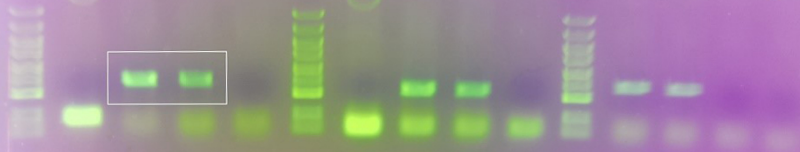

Gapdh

Supplement: Figure 3—figure supplement 2—source data 1. [file elife-88905-fig3-figsupp2-data1.zip › Figure3S2SourceData1/rawdata picture/data 5.pdf]

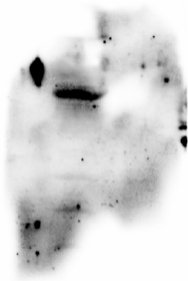

Supplement: Figure 3—figure supplement 2—source data 1. [file elife-88905-fig3-figsupp2-data1.zip › Figure3S2SourceData1/rawdata picture/data 1.pdf]

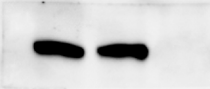

Supplement: Figure 3—figure supplement 2—source data 1. [file elife-88905-fig3-figsupp2-data1.zip › Figure3S2SourceData1/rawdata picture/data 2.pdf]

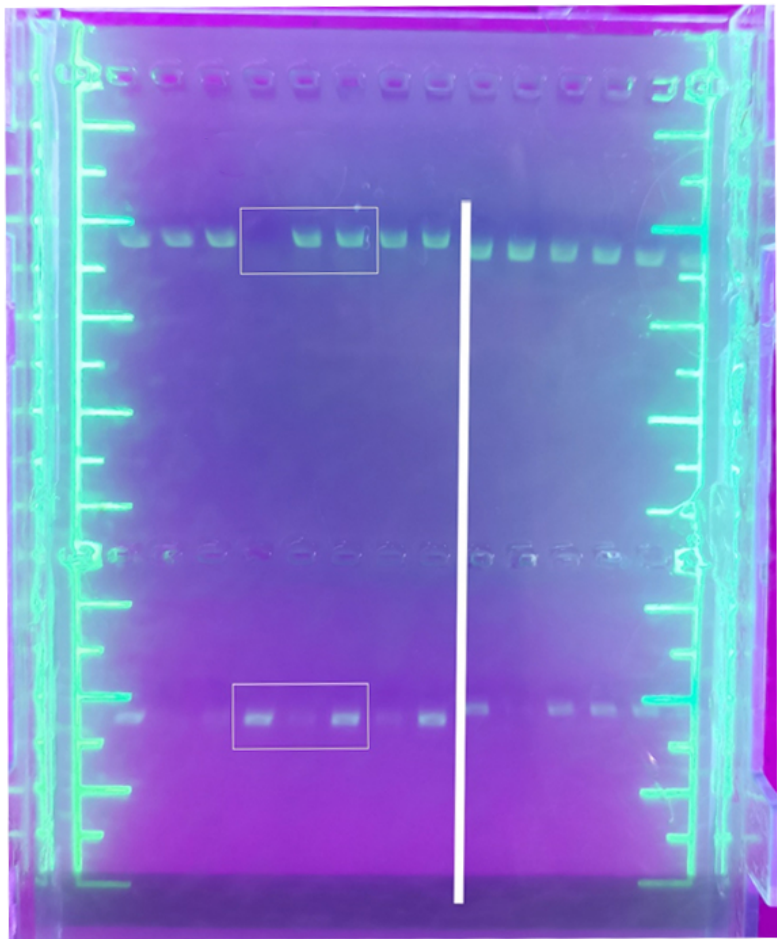

Supplement: Figure 3—figure supplement 2—source data 1. [file elife-88905-fig3-figsupp2-data1.zip › Figure3S2SourceData1/rawdata picture/data 3.pdf]

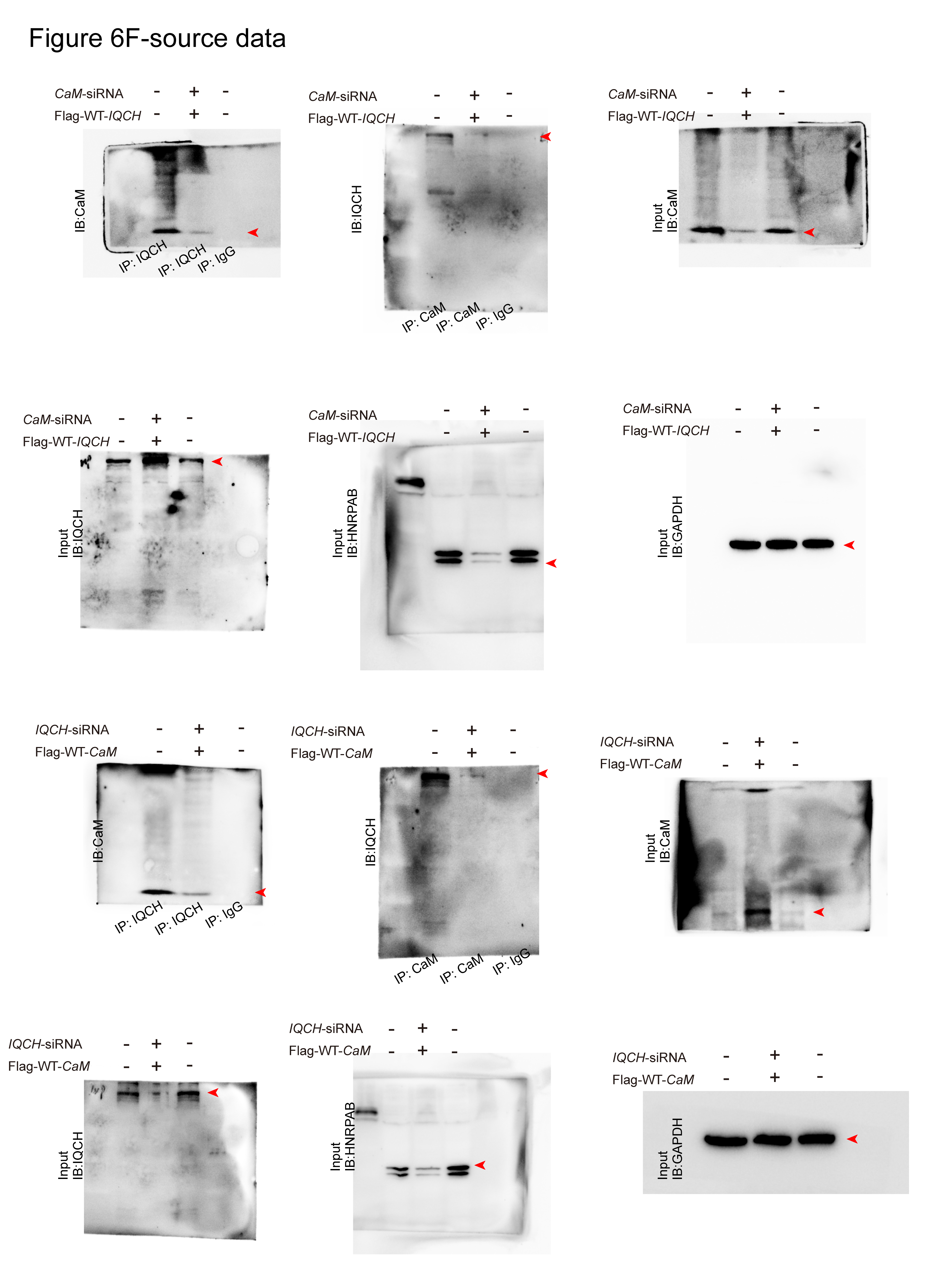

Supplement: Figure 6—source data 3. [file elife-88905-fig6-data3.zip › Figure6SourceData3/Figure 6F-source data_1.png]

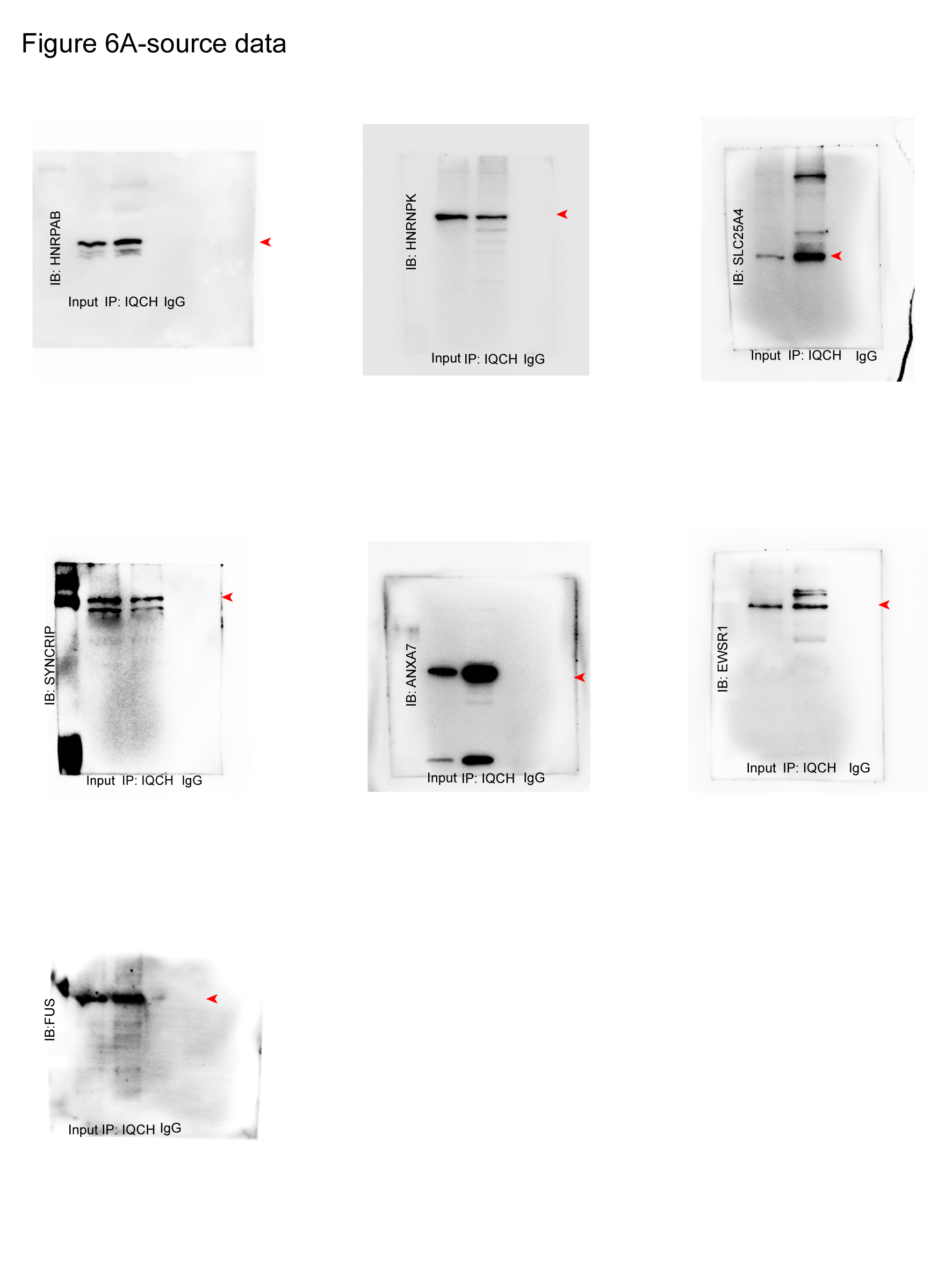

Supplement: Figure 6—source data 3. [file elife-88905-fig6-data3.zip › Figure6SourceData3/Figure 6A-source data_1.tif]

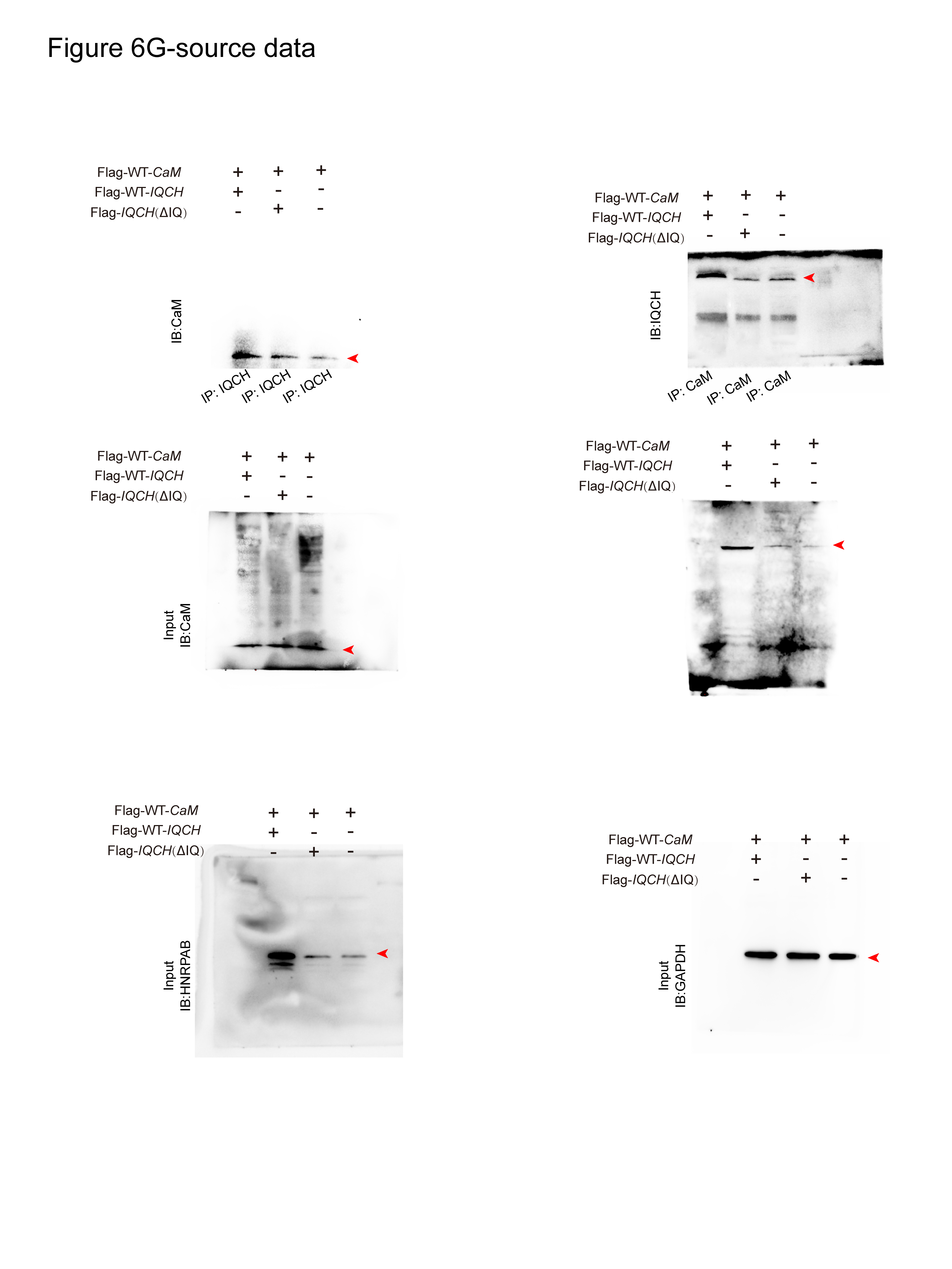

Supplement: Figure 6—source data 3. [file elife-88905-fig6-data3.zip › Figure6SourceData3/Figure 6G-source data_1.png]

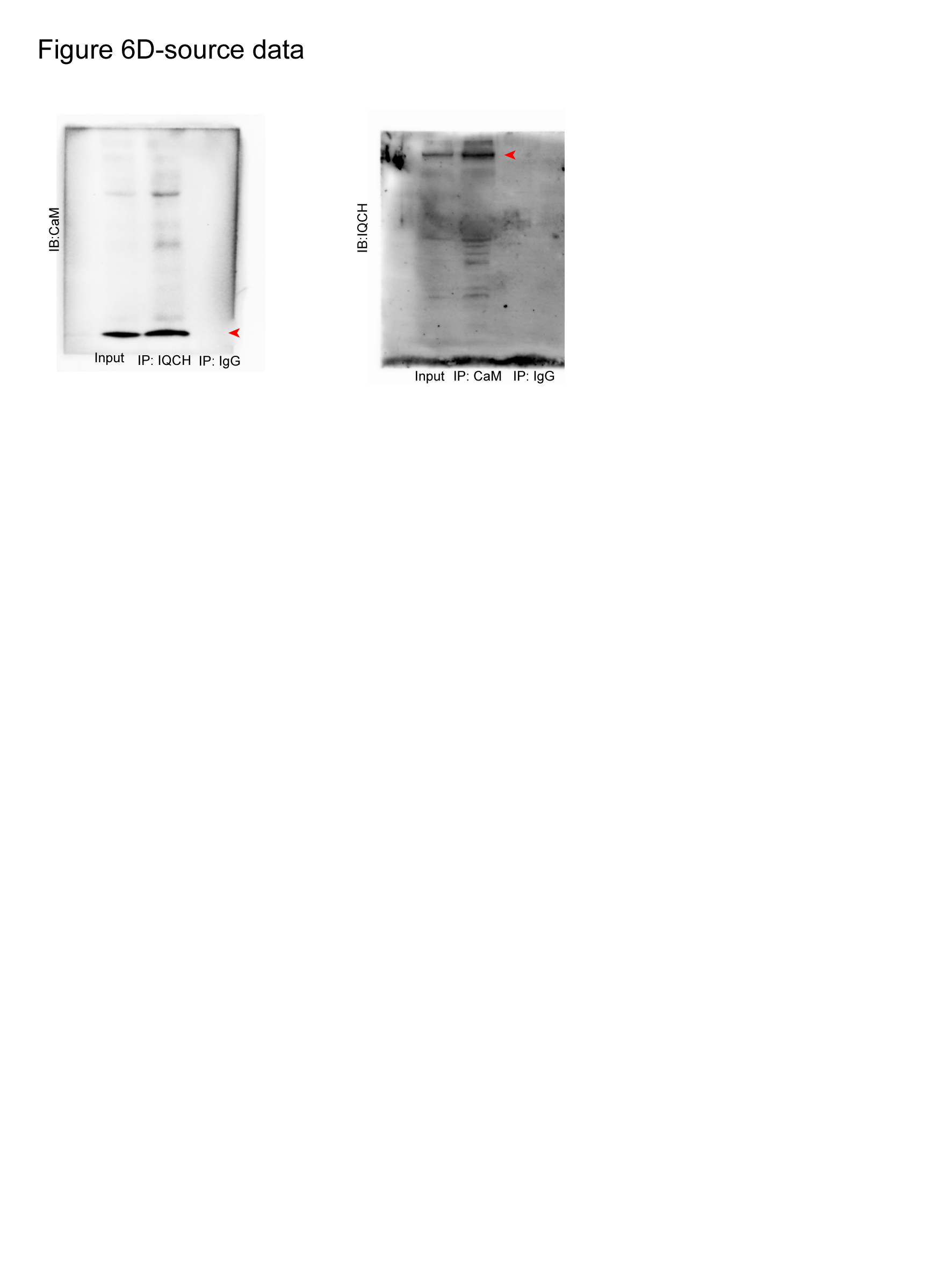

Supplement: Figure 6—source data 3. [file elife-88905-fig6-data3.zip › Figure6SourceData3/Figure 6D-source data_1.tif]

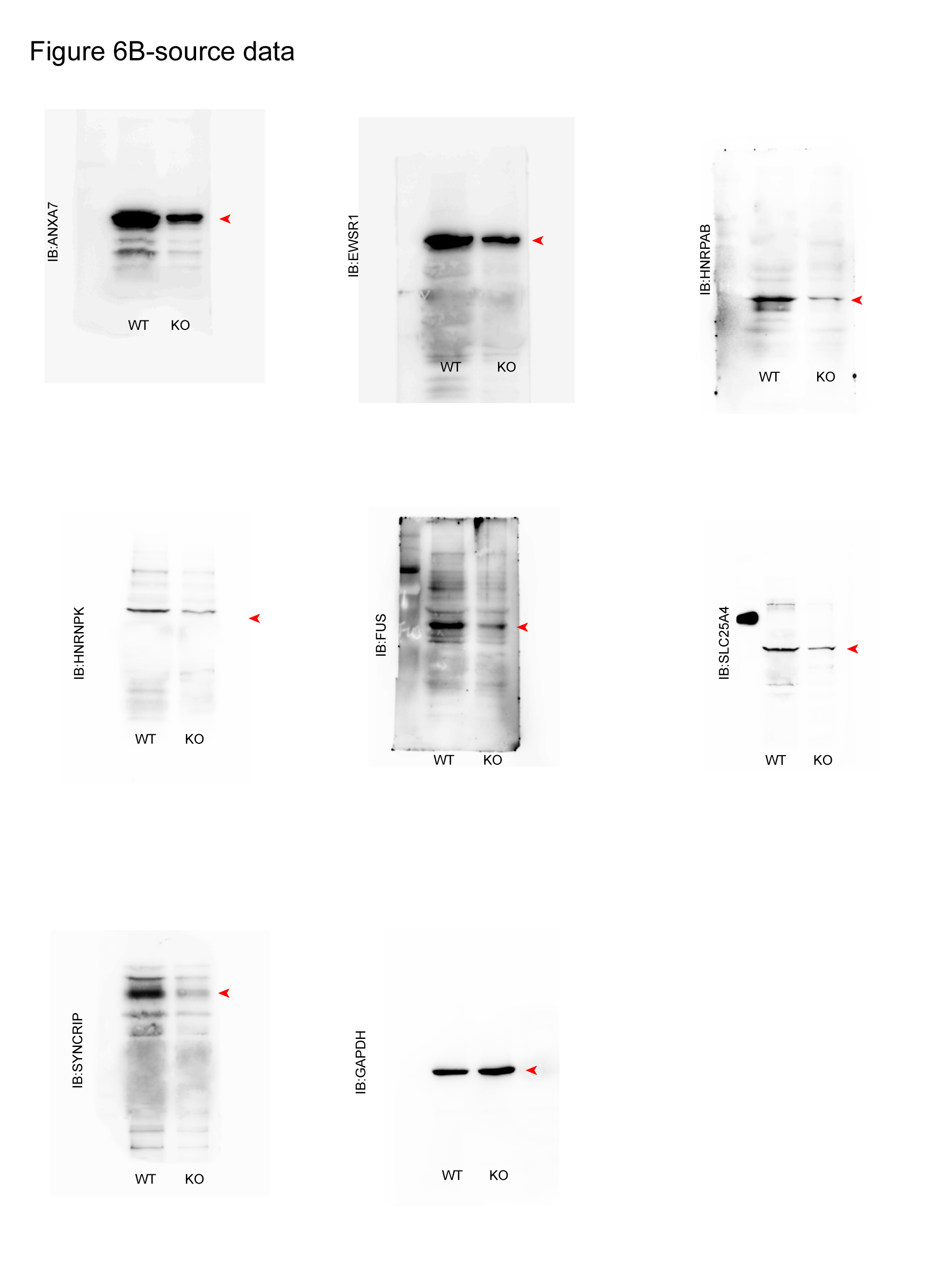

Supplement: Figure 6—source data 3. [file elife-88905-fig6-data3.zip › Figure6SourceData3/Figure 6B-source data_1.tif]

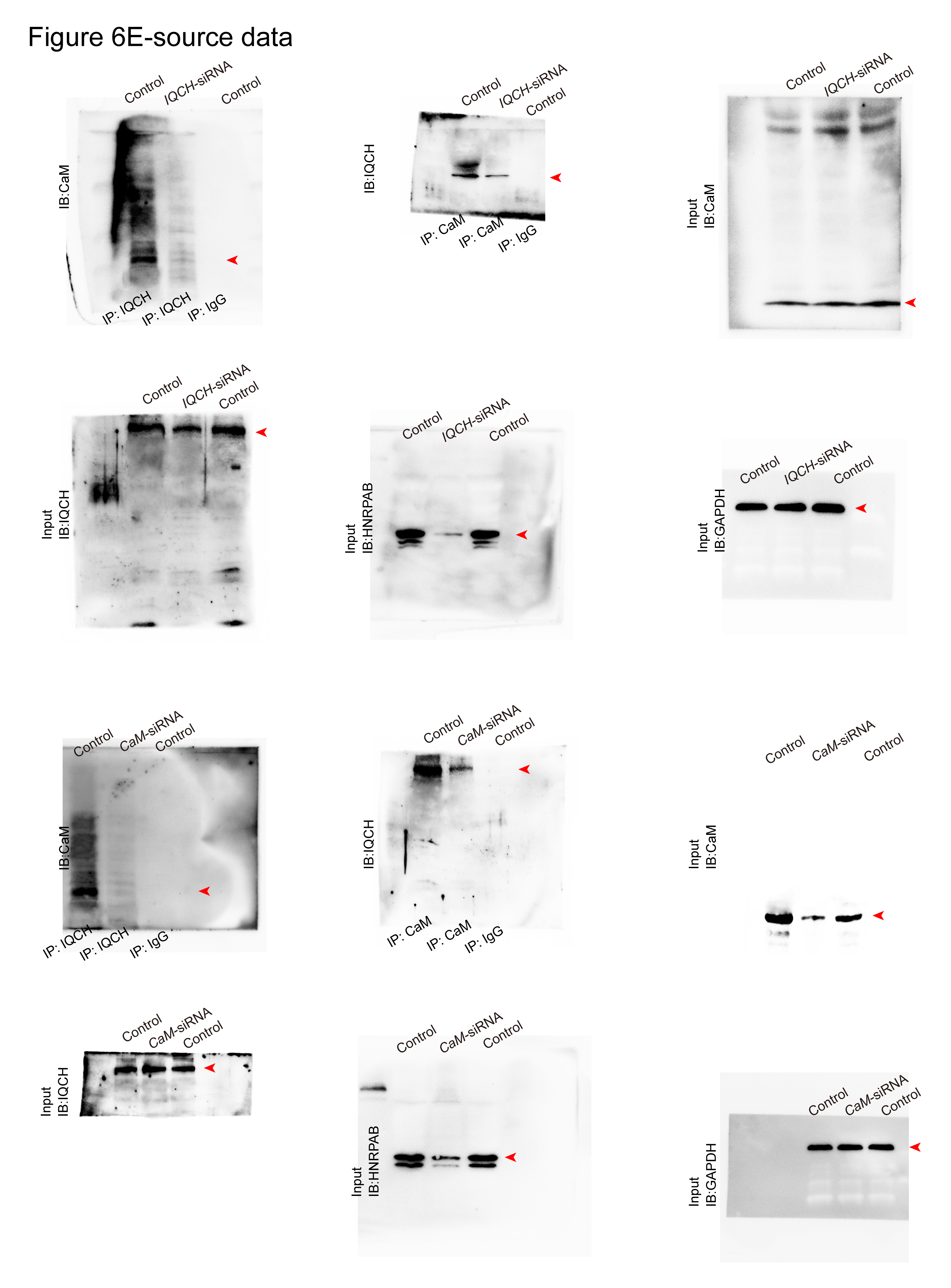

Supplement: Figure 6—source data 3. [file elife-88905-fig6-data3.zip › Figure6SourceData3/Figure 6E-source data_1.png]

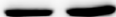

Supplement: Figure 6—source data 3. [file elife-88905-fig6-data3.zip › Figure6SourceData3/rawdata picture/Figure 6B blots/data 8.pdf]

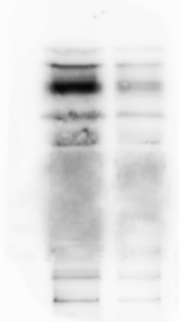

Supplement: Figure 6—source data 3. [file elife-88905-fig6-data3.zip › Figure6SourceData3/rawdata picture/Figure 6B blots/data 7.pdf]

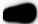

1871

1872

1873

1874

1875

1876

Supplement: Figure 6—source data 3. [file elife-88905-fig6-data3.zip › Figure6SourceData3/rawdata picture/Figure 6B blots/data 6.pdf]

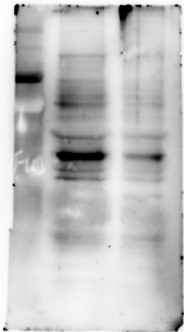

Supplement: Figure 6—source data 3. [file elife-88905-fig6-data3.zip › Figure6SourceData3/rawdata picture/Figure 6B blots/data 5.pdf]

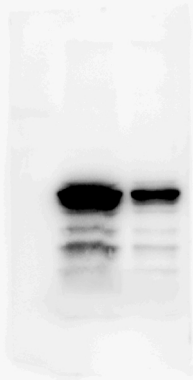

Supplement: Figure 6—source data 3. [file elife-88905-fig6-data3.zip › Figure6SourceData3/rawdata picture/Figure 6B blots/data 1.pdf]

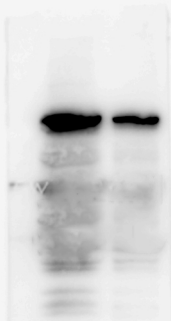

Supplement: Figure 6—source data 3. [file elife-88905-fig6-data3.zip › Figure6SourceData3/rawdata picture/Figure 6B blots/data 2.pdf]

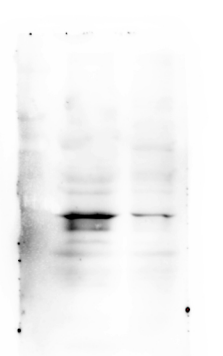

Supplement: Figure 6—source data 3. [file elife-88905-fig6-data3.zip › Figure6SourceData3/rawdata picture/Figure 6B blots/data 3.pdf]

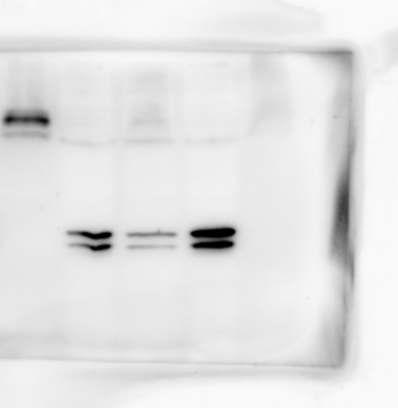

Supplement: Figure 6—source data 3. [file elife-88905-fig6-data3.zip › Figure6SourceData3/rawdata picture/Figure 6F blots/data 11.pdf]

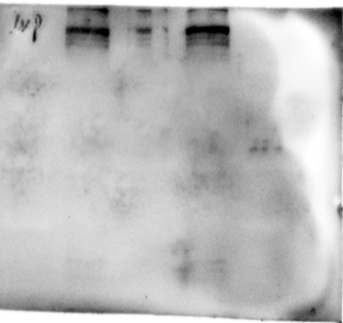

Supplement: Figure 6—source data 3. [file elife-88905-fig6-data3.zip › Figure6SourceData3/rawdata picture/Figure 6F blots/data 10.pdf]

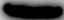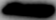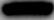

Supplement: Figure 6—source data 3. [file elife-88905-fig6-data3.zip › Figure6SourceData3/rawdata picture/Figure 6F blots/data 12.pdf]

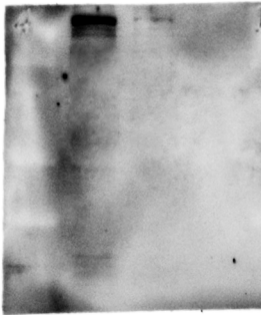

Supplement: Figure 6—source data 3. [file elife-88905-fig6-data3.zip › Figure6SourceData3/rawdata picture/Figure 6F blots/data 8.pdf]

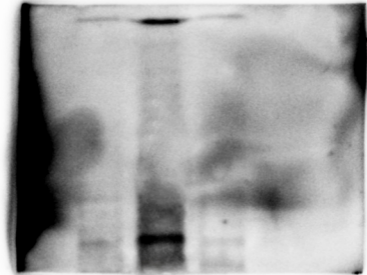

Supplement: Figure 6—source data 3. [file elife-88905-fig6-data3.zip › Figure6SourceData3/rawdata picture/Figure 6F blots/data 9.pdf]

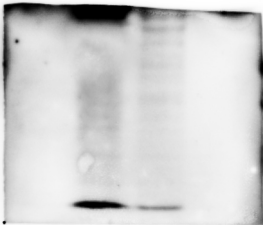

Supplement: Figure 6—source data 3. [file elife-88905-fig6-data3.zip › Figure6SourceData3/rawdata picture/Figure 6F blots/data 7.pdf]

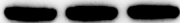

Supplement: Figure 6—source data 3. [file elife-88905-fig6-data3.zip › Figure6SourceData3/rawdata picture/Figure 6F blots/data 6.pdf]

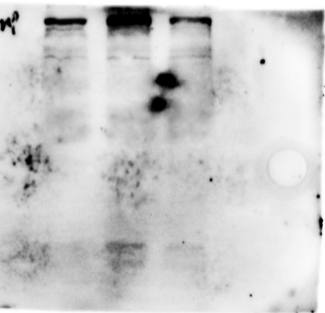

Supplement: Figure 6—source data 3. [file elife-88905-fig6-data3.zip › Figure6SourceData3/rawdata picture/Figure 6F blots/data 4.pdf]

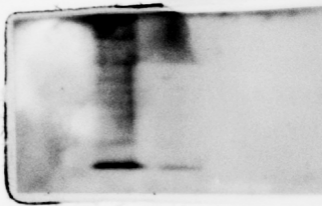

Supplement: Figure 6—source data 3. [file elife-88905-fig6-data3.zip › Figure6SourceData3/rawdata picture/Figure 6F blots/data 1.pdf]

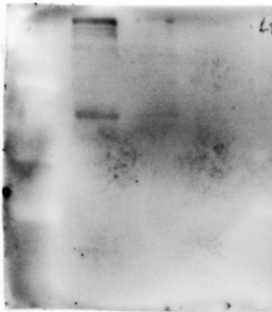

Supplement: Figure 6—source data 3. [file elife-88905-fig6-data3.zip › Figure6SourceData3/rawdata picture/Figure 6F blots/data 2.pdf]

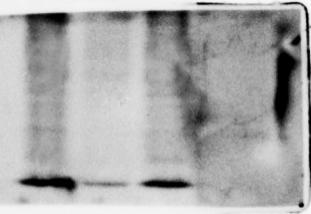

Supplement: Figure 6—source data 3. [file elife-88905-fig6-data3.zip › Figure6SourceData3/rawdata picture/Figure 6F blots/data 3.pdf]

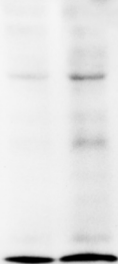

Supplement: Figure 6—source data 3. [file elife-88905-fig6-data3.zip › Figure6SourceData3/rawdata picture/Figure 6D blots/data 1.pdf]

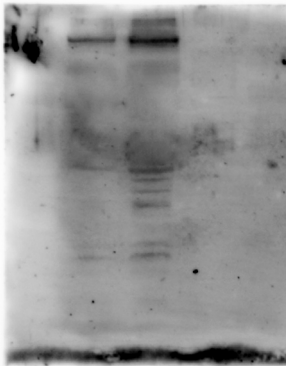

Supplement: Figure 6—source data 3. [file elife-88905-fig6-data3.zip › Figure6SourceData3/rawdata picture/Figure 6D blots/data 2.pdf]

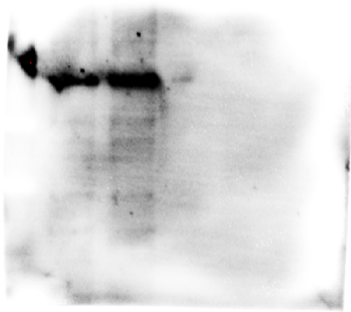

Supplement: Figure 6—source data 3. [file elife-88905-fig6-data3.zip › Figure6SourceData3/rawdata picture/Figure 6A blots/data 7.pdf]

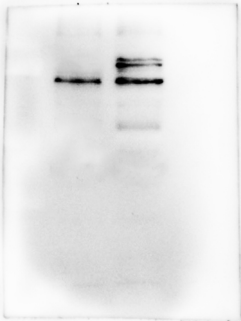

Supplement: Figure 6—source data 3. [file elife-88905-fig6-data3.zip › Figure6SourceData3/rawdata picture/Figure 6A blots/data 6.pdf]

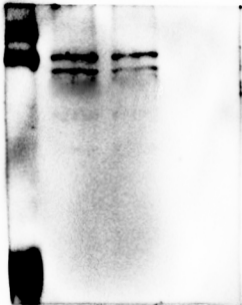

Supplement: Figure 6—source data 3. [file elife-88905-fig6-data3.zip › Figure6SourceData3/rawdata picture/Figure 6A blots/data 4.pdf]

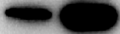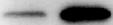

Supplement: Figure 6—source data 3. [file elife-88905-fig6-data3.zip › Figure6SourceData3/rawdata picture/Figure 6A blots/data 5.pdf]

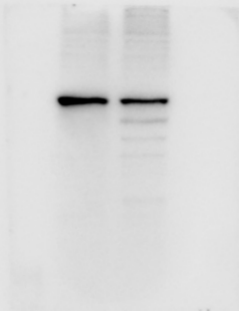

Supplement: Figure 6—source data 3. [file elife-88905-fig6-data3.zip › Figure6SourceData3/rawdata picture/Figure 6A blots/data 2.pdf]

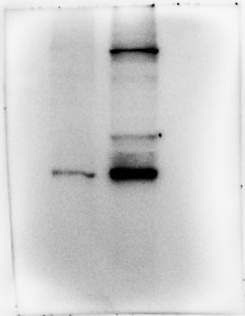

Supplement: Figure 6—source data 3. [file elife-88905-fig6-data3.zip › Figure6SourceData3/rawdata picture/Figure 6A blots/data 3.pdf]

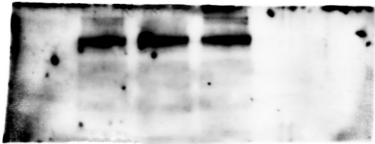

Supplement: Figure 6—source data 3. [file elife-88905-fig6-data3.zip › Figure6SourceData3/rawdata picture/Figure 6E blots/data 10.pdf]

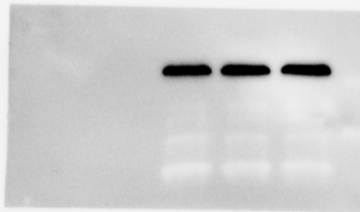

Supplement: Figure 6—source data 3. [file elife-88905-fig6-data3.zip › Figure6SourceData3/rawdata picture/Figure 6E blots/data 12.pdf]

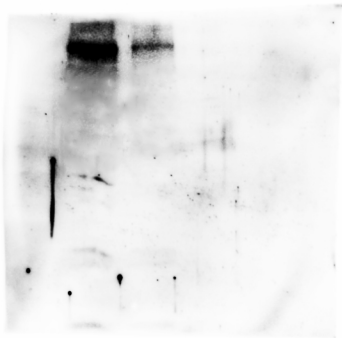

Supplement: Figure 6—source data 3. [file elife-88905-fig6-data3.zip › Figure6SourceData3/rawdata picture/Figure 6E blots/data 8.pdf]

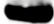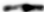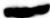

Supplement: Figure 6—source data 3. [file elife-88905-fig6-data3.zip › Figure6SourceData3/rawdata picture/Figure 6E blots/data 9.pdf]

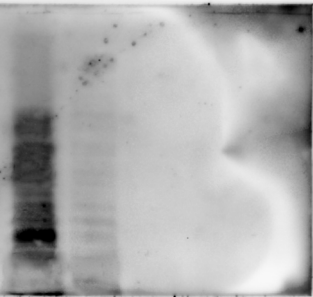

Supplement: Figure 6—source data 3. [file elife-88905-fig6-data3.zip › Figure6SourceData3/rawdata picture/Figure 6E blots/data 7.pdf]

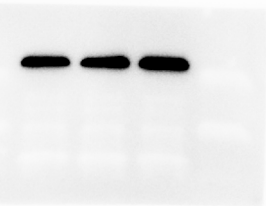

Supplement: Figure 6—source data 3. [file elife-88905-fig6-data3.zip › Figure6SourceData3/rawdata picture/Figure 6E blots/data 6.pdf]

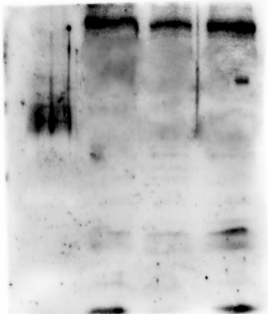

Supplement: Figure 6—source data 3. [file elife-88905-fig6-data3.zip › Figure6SourceData3/rawdata picture/Figure 6E blots/data 4.pdf]

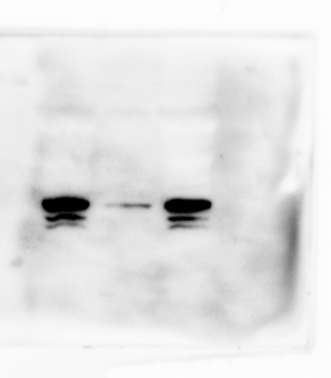

Supplement: Figure 6—source data 3. [file elife-88905-fig6-data3.zip › Figure6SourceData3/rawdata picture/Figure 6E blots/data 5.pdf]

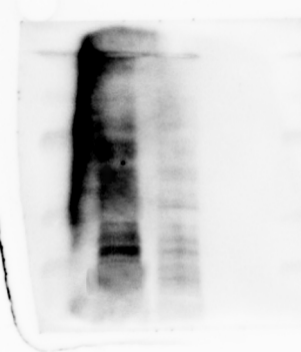

Supplement: Figure 6—source data 3. [file elife-88905-fig6-data3.zip › Figure6SourceData3/rawdata picture/Figure 6E blots/data 1.pdf]

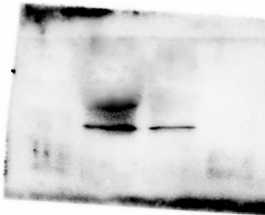

Supplement: Figure 6—source data 3. [file elife-88905-fig6-data3.zip › Figure6SourceData3/rawdata picture/Figure 6E blots/data 2.pdf]

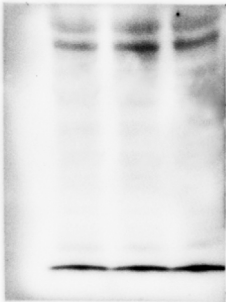

Supplement: Figure 6—source data 3. [file elife-88905-fig6-data3.zip › Figure6SourceData3/rawdata picture/Figure 6E blots/data 3.pdf]

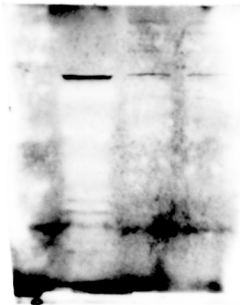

Supplement: Figure 6—source data 3. [file elife-88905-fig6-data3.zip › Figure6SourceData3/rawdata picture/Figure 6G blots/data 4.pdf]

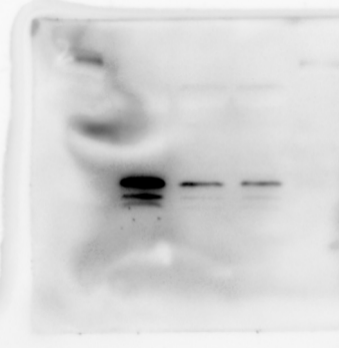

Supplement: Figure 6—source data 3. [file elife-88905-fig6-data3.zip › Figure6SourceData3/rawdata picture/Figure 6G blots/data 5.pdf]

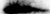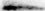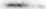

Supplement: Figure 6—source data 3. [file elife-88905-fig6-data3.zip › Figure6SourceData3/rawdata picture/Figure 6G blots/data 1.pdf]

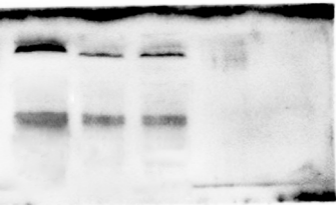

Supplement: Figure 6—source data 3. [file elife-88905-fig6-data3.zip › Figure6SourceData3/rawdata picture/Figure 6G blots/data 2.pdf]

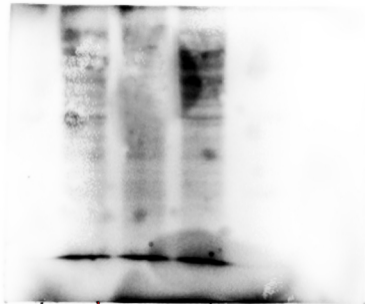

Supplement: Figure 6—source data 3. [file elife-88905-fig6-data3.zip › Figure6SourceData3/rawdata picture/Figure 6G blots/data 3.pdf]
