# Supplementary material for: Deficiency of IQCH causes male infertility in humans and mice
Source: eLife. 2024 Jul 19;12:RP88905. doi: 10.7554/eLife.88905 (PMC11259432; doi:10.7554/eLife.88905)

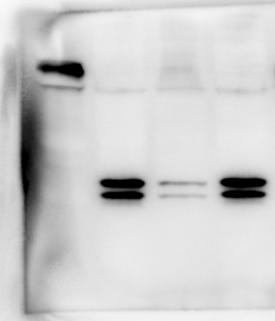

Supplement: Figure 6—source data 3. [file elife-88905-fig6-data3.zip › Figure6SourceData3/rawdata picture/Figure 6F blots/data 5.pdf]
